# The Association of Serum and Dietary Magnesium with Depressive Symptoms

**DOI:** 10.3390/nu15030774

**Published:** 2023-02-02

**Authors:** Ming-Hui Chou, Yen Kuang Yang, Jung-Der Wang, Chung-Ying Lin, Sheng-Hsiang Lin

**Affiliations:** 1 Department of Public Health, College of Medicine, National Cheng Kung University, Tainan 704, Taiwan; 2 Department of Psychiatry, National Cheng Kung University Hospital, College of Medicine, National Cheng Kung University, Tainan 704, Taiwan; 3 Institute of Behavioral Medicine, College of Medicine, National Cheng Kung University, Tainan 704, Taiwan; 4 Department of Psychiatry, Tainan Hospital, Ministry of Health and Welfare, Tainan 700, Taiwan; 5 Department of Occupational Medicine, National Cheng Kung University Hospital, College of Medicine, National Cheng Kung University, Tainan 704, Taiwan; 6 Institute of Allied Health Sciences, College of Medicine, National Cheng Kung University, Tainan 704, Taiwan; 7 Biostatistics Consulting Center, National Cheng Kung University Hospital, College of Medicine, National Cheng Kung University, Tainan 704, Taiwan; 8 Institute of Clinical Medicine, College of Medicine, National Cheng Kung University, 35 Siaodong Rd., Tainan 704, Taiwan

**Keywords:** serum magnesium, dietary magnesium intake, depression, depressive symptoms, chronic latent magnesium deficiency (CLMD), Nutrition and Health Survey in Taiwan (NAHSIT), 5-item Brief Symptom Rating Scale (BSRS-5)

## Abstract

Depression is a leading cause of the global burden of disease and has a multifactorial etiology that includes nutrients. Magnesium status has been associated with depression with inconclusive results. The impact of chronic latent magnesium deficiency (CLMD, 0.75 ≤ serum magnesium < 0.85 mmol/L) on depression has not yet been investigated. We assessed the association between serum magnesium levels/dietary magnesium intake and depressive symptoms by analyzing nationally representative data from Taiwan (Nutrition and Health Survey in Taiwan, NAHSIT). We used the 5-item Brief Symptom Rating Scale to measure depressive symptoms. Subgroup analysis by sex was also performed. Serum magnesium levels had a low correlation with dietary magnesium intake. Higher serum magnesium levels were associated with lower depressive scores and a lower risk of depressive symptoms, but dietary magnesium intake showed no association. Sex differences were found. Compared with subjects with serum magnesium <0.75 mmol/L, those with ≥0.85 mmol/L had lower depressive scores. In conclusion, serum magnesium was inversely associated with depressive symptoms, but dietary magnesium intake was not. Subjects with CLMD showed similar depressive scores and were at a similar risk of depressive symptoms to those with serum magnesium < 0.75 mmol/L. CLMD should be considered while assessing the association between magnesium status and depressive symptoms.

## 1. Introduction

Depression is a serious mental disorder affecting the quality of life. It is a major contributor to the overall global burden of disease and the top cause of years lived with disability (YLD) [1]. Depression accounts for 5.45% of YLD in all non-communicable and communicable conditions, and is a leading cause of non-communicable disability [2]. In the United States, depression is the second leading cause of disability [3] and is a contributor to the burden allocated to suicide and ischemic heart disease [4]. A similar scenario exists in Asia. For example, in Taiwan, depression accounted for 4.3% of YLD in 2019 and grows by an annual rate of 1.2%. Meanwhile, the National Health Insurance spends approximately USD 1.342 billion annually on antidepressants for depression treatment.

Magnesium is the fourth most abundant mineral nutrient in the human body; it plays a fundamental role in blood pressure regulation, glucose metabolism, stress coping, and neurotransmitter regulation in the central nervous system. The relation between depression and magnesium may be based on the following mechanism: Depression is associated with the dysregulation of the hypothalamic–pituitary–adrenal (HPA) axis. Magnesium deficiency triggers the release of the corticotropin-releasing hormone, consequently increasing the level of the adrenocorticotropin hormone (ACTH) [5]. Stress diathesis and monoamine dysregulation have been widely recognized as important mechanisms in the development of depression [5] and are also dysfunctional during HPA excitability. N-methyl-d-aspartate (NMDA) receptors play a fundamental role in the pathogenesis of depression [6,7]. The NMDA receptors are activated when glutamates bind and return to their resting state with the binding of magnesium. NMDA-coupled calcium channels are inclined to activate in a magnesium-deficient state, which causes neuronal damage [8].

The relationship between magnesium and depression has been examined in previous reports, both with serum magnesium and magnesium dietary intake [9,10,11,12,13,14,15]. Defining a perfect indicator that appropriately reflects the magnesium status in the human body is challenging. Due to the sophisticated biological mechanism of absorption and the sensitive compartmental handling of magnesium under different circumstances, dietary magnesium intake does not represent the body’s magnesium functional status [16]. Serum magnesium only makes up ~1% of total body magnesium and cannot represent the intracellular magnesium status. However, because we lack a more direct, specific and easily available biomarker, serum magnesium is currently the most acceptable laboratory test for evaluating clinical magnesium status, and has been widely adopted as an indicator of magnesium status to test the association between magnesium and depression [17,18,19,20]. The association between dietary magnesium intake and serum magnesium, and the risk of depression is currently inconclusive. Previous studies only demonstrated the association with serum or dietary magnesium, but never in the same study sample to test the differentiation between these two indicators. The association of serum and dietary magnesium, at the same time with other diseases, such as diabetes mellitus (DM) and hypertension, is shown in previous studies [10,21,22,23].

Hypomagnesemia is associated with manifestations that vary from asymptomatic to severe. Clinical manifestations include endocrine abnormalities (insulin resistance and diabetes mellitus), cardiovascular abnormalities (hypertension, arrhythmia), neuromuscular–central nervous system symptoms (tremor, headache, hyperexcitability), and others. Other than severe diseases and overt symptoms, people can experience asymptomatic hypomagnesemia or chronic latent magnesium deficiency (CLMD) at serum magnesium levels higher than 0.6 mmol/L. CLMD is a condition of chronic subtle negative magnesium balance, which causes affected individuals to be more vulnerable to diseases, while the serum magnesium levels fall into the lower half of the reference interval (0.75–0.85 mmol/L). Possible reasons for CLMD include a decreased dietary intake, decreased absorption through the gastrointestinal tract, and increased renal loss [24]. Decreased dietary intake was the most important reason and has attracted attention due to changes in eating habits, such as the increased intake of processed and fast food, which contain less magnesium than whole foods [25,26]. However, illness, drug use, and chronic stress, which also increased the depletion of magnesium, are important as well. The long-term negative balance between magnesium absorption and depletion resulted in decreased serum magnesium levels. To respond to the excessive loss of magnesium, magnesium storage, mainly from bone, is released to maintain the circulating magnesium within a normal range. Those with CLMD thus appear to have serum magnesium levels in the “normal” range, but the actual serum magnesium functionality might be inadequate; this impairs the protective function of magnesium against diseases, including depression. Nevertheless, such a deficiency may be neglected due to the examination results falling in the normal levels of current reference ranges. The suggested reference range was based on measuring serum magnesium in “healthy” individuals in the NHANES (1974), who were not aware of their CLMD condition [27]. A call for a standardized and evidence-based normal interval for serum magnesium has emerged in recent decades [28,29,30]. The above studies focus mainly on physical disease, and thus the impact on CLMD and depressive symptoms has not yet been thoroughly investigated.

In order to demonstrate the discriminative effect of serum magnesium level and dietary magnesium intake on depressive symptoms, we conducted a study to examine the relationship between serum magnesium level and dietary magnesium intake, and the association with depressive symptoms in a community-based national representative adult population in Taiwan. To further define an optimal serum magnesium level against depressive symptoms, we also discuss different risks based on different serum magnesium stratifications.

## 2. Materials and Methods

### 2.1. Data Source

The Nutrition and Health Survey in Taiwan (NAHSIT) is a government-sponsored nationally representative cross-sectional nutrition survey conducted by the Ministry of Health and Welfare in Taiwan, beginning in 1993. This study used the NAHSIT data collected from 2005 to 2008 (NAHSIT 2005–2008). NAHSIT 2005–2008 used a stratified three-staged probability sampling design. More details about the design and sample characteristics of the NAHSIT 2005–2008 have been described in a previously published paper [31]. The survey included two phases: (1) a questionnaire and (2) physical examinations. Each subject that was sampled and considered successful completed sociodemographic information, household information, the 24-hour dietary recall in the household interview, and was then invited to participate in the health exam. A total of 4615 community-dwelling Taiwanese citizens, aged 20 years and above, were included in the analysis. The exclusion criteria of this study included participants who did not provide any of the following data: (1) BSRS-5 (5-item Brief Symptom Rating Scale), (2) 24-hour diet recall, (3) sociodemographic information, or (4) blood biochemical parameters. After exclusion, 2193 participants were included in this study. The approval to conduct this study was granted by the National Cheng Kung University Research Ethics Review Board (Protocol #B-EX-110-014).

### 2.2. BSRS-5 (5-Item Brief Symptom Rating Scale)

We used the BSRS-5, a self-administered questionnaire, to assess the participants’ psychological health status. The BSRS-5 was derived from the Symptom Checklist-90-Revised (SCL-90-R) and has been validated with excellent validity and reliability in both medical and community settings in Taiwan. The BSRS-5 is used to measure five different dimensions: (1) anxiety (feeling tense or being keyed up), (2) depression (feeling blue or sad), (3) hostility (feeling easily annoyed or irritated), (4) interpersonal hypersensitivity (feeling inferior to others), and (5) additional symptoms (having trouble falling asleep). The item responses ranged from 0 to 4 (0 = not at all; 1 = a little bit; 2 = moderately; 3 = quite a bit; and 4 = extremely). The total score ranged from 0 to 20; a higher score indicated a higher risk of psychological disorder.

### 2.3. Magnesium

Two types of magnesium exposure were assessed: serum magnesium (mg/dL) and dietary magnesium intake (mg). Serum magnesium concentration was measured by the colorimetric method using a Roche Cobas Integra 800 (Roche, Basel, Switzerland). To conduct the colorimetric method, an alkaline complex with absorption at 520 nm was formed. The formation of the alkaline complex is positively associated with the level of serum magnesium. Calcium interference was avoided by using Glycoletherdiamine-N,N,N′,N′-tetraacetic acid (GEDTA). Dietary magnesium intake was assessed using the 24-hour dietary recall questionnaire [32].

### 2.4. Covariates

Based on previous studies on magnesium and psychological distress, a wide range of covariates were included in the analysis. We included the following potential sociodemographic confounding variables: age (continuous, range 20–101), sex, education level (none, below senior high school [SHS], SHS, and above SHS), and income level (no income, 20,000 New Taiwan dollars [NTD] or lower, NTD 20,001 to 50,000, NTD 50,001 to 70,000, and NTD 70,001 or greater).

Physical activity status was determined by several questions regarding whether the participants were engaged in the following activities regularly: walking, running, hiking, folk dancing, aerobic dancing, swimming, and bicycling. Participants were grouped into three levels: (1) no, (2) yes—walking, and (3) yes—more than walking. Alcohol use and smoking status were grouped into non-drinker, former drinker, and current drinker and non-smoker, former smoker, and current smoker, respectively. The history of comorbidities was determined by the participants’ self-reported data regarding whether they had been diagnosed with hypertension, kidney disease, and diabetes mellitus by doctors (yes versus no). We obtained the total energy intake from the 24-hour dietary recall questionnaire. Serum c-reactive protein (CRP) levels (mg/L) were grouped into three clinically relevant categories: (1) CRP < 1, (2) 1 ≤ CRP < 3, and (3) CRP ≥ 3. Calcium was also included for its interaction with magnesium.

### 2.5. Statistical Analysis

Differences in covariates by BSRS total scores and the scores of each BSRS item were tested using *t*-tests or ANOVAs for categorical variables, and simple linear regression analysis for continuous variables. Pearson correlations were estimated between dietary magnesium and serum magnesium after removing the 1% extreme values. We conducted a multiple linear regression analysis and logistic regression to explore the associations of BSRS-5, both total scores and the scores for each item, and magnesium and the analyses were adjusted for covariates. Subgroup analysis was conducted by sex. All analyses were conducted using SAS version 9.4 (SAS Institute, Cary, NC, USA).

## 3. Results

### 3.1. Correlations between Dietary Magnesium and Serum Magnesium

**Figure 1:**
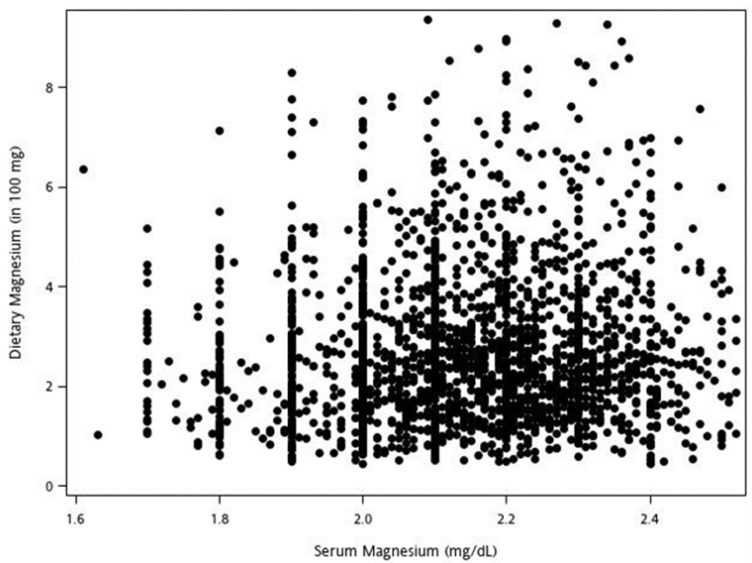
Scatter plot of dietary magnesium versus serum magnesium (correlation: r = 0.073, *p* value ≤ 0.001).

Pearson correlations were used to estimate correlations between dietary magnesium and serum magnesium. We excluded the highest and lowest 1% extreme values to preclude the effect of the outlier. The correlation coefficient was 0.073. The low correlation implies that a higher dietary magnesium intake does not necessarily represent higher serum magnesium concentrations (Figure 1).

### 3.2. Demographic Variables

The characteristics of the included subjects are listed in Table 1. In total, 1124 (51.25%) participants in this study were women. The education level of 934 (42.59%) participants in this study was below SHS, and more than 60% of the participants had an income level of NTD 20,000 or lower. Regarding behavior-related factors, 977 (44.55%) participants had no exercise habits, 1183 (53.94%) were non-drinkers and 1490 (67.94%) were non-smokers. Most of the participants had a CRP level < 1 mg/L (1041; 47.47%), 188 (8.57%) had DM, 528 (24.08%) had hypertension and 40 (1.82%) had kidney disease. The mean age was 53.36 (±17.36) years old; the mean dietary magnesium (Mg) intake was 278 (±185) mg/day; the mean serum Mg was 2.14 (±0.19) mg/dL; the mean dietary calcium (Ca) intake was 611 (±527) mg/day; the mean serum Ca was 9.18 (±0.43) mg/dL; and the mean BSRS-5 total score was 1.86 (±2.6) (Table 1).

### 3.3. Association of Magnesium and BSRS-5

The results of the multivariate regression showed that serum Mg was negatively correlated with BSRS score (β = −0.85, 95% CI −1.43 to −0.27), insomnia (β = −0.26, 95% CI −0.48 to −0.03), depression (β = −0.26, 95% CI −0.41 to −0.10), and inferiority (β = −0.17, 95% CI −0.30 to −0.04). After adjusting for age, sex, educational levels, income levels, smoking status, alcohol use, physical activity status, hypertension, diabetes mellitus, kidney disease, c-reactive protein, and total energy intake (per 1000 kcal), the result still showed a negative association with depressive scores (β = −0.18, 95% CI −0.34 to −0.02). Further, for model 2, adjusted for all the variables in model 1 plus calcium (Ca), the result showed a negative association with depressive scores (β = −0.16, 95% CI −0.34 to −0.03). However, dietary Mg was not significantly associated with the depressive, or any other scores, of BSRS-5 items in either the simple model or the adjusted models (Table 2).

We also categorized the outcomes as binary results. Those with a BSRS-5 total score ≥ 6 were classified as positive for psychological distress. In every single item of BSRS-5, scores ≥ 2 were classified as positive for symptoms. The results of the multivariate logistic regression showed that serum Mg was protectively correlated with BSRS score (OR = 0.436, 95% CI 0.203 to 0.938), insomnia (OR = 0.518, 95% CI 0.287 to 0.933), depression (OR = 0.315, 95% CI 0.135 to 0.733), and inferiority (OR = 0.213, 95% CI 0.076 to 0.599). After adjusting for age, sex, educational levels, income levels, smoking status, alcohol use, physical activity status, hypertension, diabetes mellitus, kidney disease, c-reactive protein, and total energy intake (per 1000 kcal), the result showed a protective effect against depression (OR = 0.378, 95% CI 0.155 to 0.924) and inferiority (OR = 0.305, 95% CI 0.102 to 0.912). Model 2 further adjusted all the variables in model 1, plus Ca, and was marginally significant for depression (OR = 0.420, 95% CI 0.170 to 1.035). However, dietary Mg was not significantly associated with any item of BSRS-5 in either the simple or adjusted models (Table 3).

### 3.4. Sex Stratification for Analysis of BSRS-5 Versus Serum Magnesium

For men, the analysis results show that serum magnesium was negatively associated with BSRS total score (β = −1.79, 95% CI −2.58 to −0.99), insomnia (β = −0.76, 95% CI −1.07 to −0.45), anxiety (β = −0.25, 95% CI −0.42 to −0.07), depression (β = −0.39, 95% CI −0.60 to −0.18), and inferiority (β = −0.23, 95% CI −0.42 to −0.05) in the simple model (Table 3). Further, model 1 adjusted the risk factors, including age, educational levels, income levels, smoking status, alcohol use, physical activity status, hypertension, diabetes mellitus, kidney disease, c-reactive protein, and total energy intake (per 1000 kcal). Results consistent with the simple model were observed: there was a significant negative impact on BSRS total score (β = −1.49, 95% CI −2.31 to −0.67), insomnia (β = −0.61, 95% CI −0.93 to −0.28), anxiety (β = −0.25, 95% CI −0.43 to −0.07), and depression (β = −0.35, 95% CI −0.58 to −0.13) (Table 3). Derived from model 1, Ca was included to establish model 2, which showed consistent results with model 1 (a significant negative impact on BSRS total score (β = −1.56, 95% CI −2.39 to −0.73), insomnia (β = −0.64, 95% CI −0.97 to −0.32), anxiety (β = −0.25, 95% CI −0.43 to −0.07), and depression (β = −0.36, 95% CI −0.59 to −0.13)) (Table 3). In women, only a positive association with insomnia was observed in the adjusted models (Table 4).

### 3.5. Stratification Analysis of the Relation between Serum Magnesium and Depression

**Figure 2:**
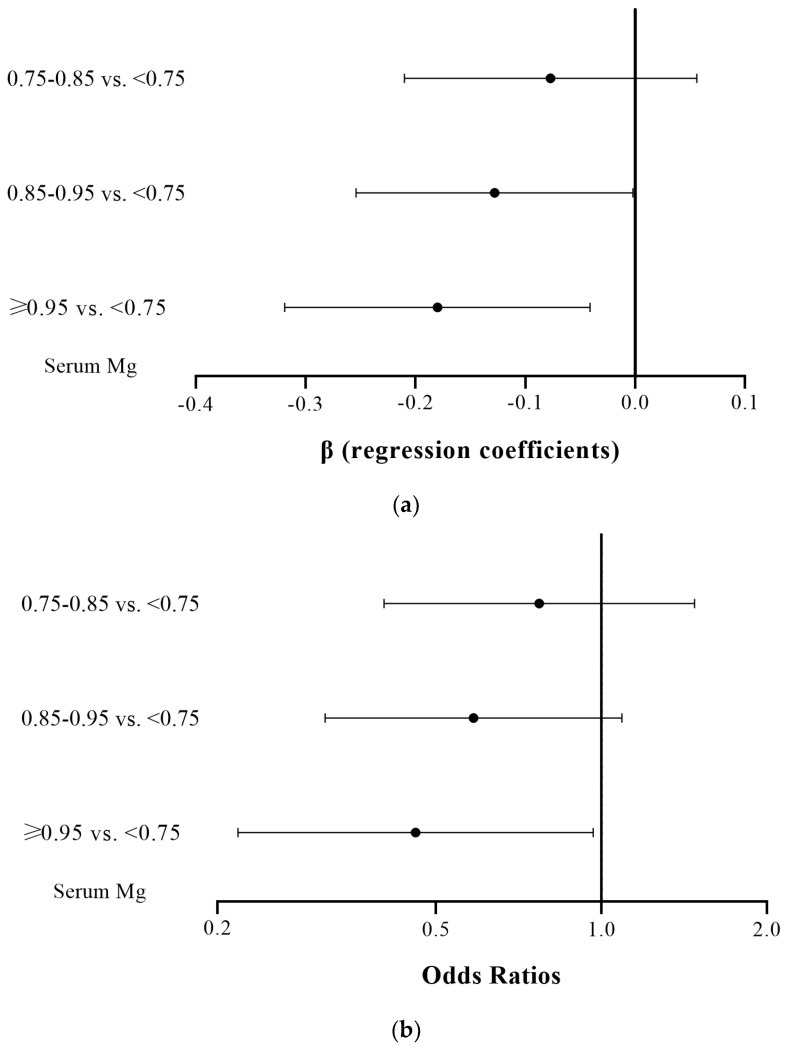
(**a**) Adjusted β (regression coefficient) of depressive symptoms versus serum magnesium (Mg). The reference group was serum Mg < 0.75 mmol/L. From top to bottom, β value for the three comparisons were [−0.077 (95% CI −0.210–0.056)], [−0.128 (95% CI −0.254–−0.002)], and [−0.180 (95% CI −0.319–−0.041)]. (**b**) Adjusted odds ratios of depressive symptoms versus serum magnesium (Mg). The reference group was serum Mg < 0.75 mmol/L. From top to bottom, the odds ratio for the three comparisons were [0.770 (95% CI 0.402–1.477)], [0.585 (95% CI 0.314–1.090)], and [0.459 (95% CI 0.218–0.966)].

Serum magnesium was further stratified into four groups (<0.75 mmol/L; ≥0.75 mmol/L and <0.85 mmol/L; ≥0.85 mmol/L and <0.95 mmol/L; ≥0.95 mmol/L), and the outcome variables for depression were divided into binary and continuous variables for regression analysis. The adjusted covariates included age, educational levels, income levels, smoking status, alcohol use, physical activity status, hyper-tension, diabetes mellitus, kidney disease, c-reactive protein, total energy intake (per 1000 kcal) and calcium. A dose–response result can be observed in Figure 2, with higher serum magnesium concentrations having a higher protective effect against depressive symptoms. The relevant data are shown in the footnotes of Figure 2a,b.

## 4. Discussion

After adjusting for potential confounders and applying two different statistical models, we consistently found serum magnesium to be inversely associated with depressive symptoms, but dietary magnesium intake was not. To our knowledge, this is the first study to examine the association of serum magnesium concentration and dietary magnesium intake with the risk and severity of depressive symptoms with a dose–response effect in community-dwelling populations. This study also shows that subjects with CLMD have similar risk and symptom severity to those with hypomagnesemia. Our results also show that serum magnesium had a low correlation with dietary magnesium. Serum magnesium levels were negatively associated with depressive symptoms in the overall sample, among men, but not among women.

There was a statistically significant association between serum magnesium and depressive symptoms, both in linear and logistic regression. For every one mg/dL increase in the serum magnesium concentration, the score of depressive symptoms decreased by 0.18, with an OR of 0.378. Further adjusting calcium into the model still showed that the score of depressive symptoms decreased by 0.16, with an OR of 0.420 (marginally significant). However, there was no such association between dietary magnesium intake and depressive symptoms. Similar to previous studies investigating the relationship between magnesium status and diabetes mellitus, there was no association between dietary magnesium intake and diabetes mellitus, while there was an inverse association between serum magnesium and diabetes mellitus [21,22]. Similar studies assessing the association with hypertension were conducted, but the conclusions were relatively inconsistent [10,23]

Why should low serum magnesium levels, but not a low dietary magnesium intake, be associated with depressive symptoms? First, compared with serum magnesium, the measurement of dietary intake is less precise and could have resulted in a type 2 error. Second, dietary magnesium intake has to pass through complicated pathways of absorption, depletion and storage to meet the changing daily body needs. Thus, the same magnesium intake could contribute differently to the body’s magnesium state from one day to another, and dietary magnesium intake might not represent the functional magnesium status within the body. In contrast, serum magnesium levels reflect the dynamic balance between different pathways of compartmental handling of whole-body magnesium, and could represent the magnesium status more directly. This is congruent with a low correlation between a dietary magnesium intake and serum magnesium levels in our study (r = 0.073), similar to that found in the Atherosclerosis Risk in Communities (ARIC) cohort (r = 0.06) [33]. Nevertheless, this does not preclude the possibility that pharmacological doses of magnesium supplements might influence serum magnesium levels. We hypothesize that, if the aim was to prevent the risk caused by hypomagnesemia, the effect of dietary magnesium intake/supplement probably needs to achieve adequate magnesium functionality through the correction of serum magnesium levels.

Sex differences were observed in our results. Sex hormones and menstruation cycles in females could explain why the association was found in men but not in women. The fluctuation of estrogen and progesterone during different menstruation phases increases the vulnerability of women to depressive symptoms [34]. Furthermore, serum magnesium levels have been reported to vary during different phases of the menstrual cycle with the effect of sex hormones [35]. Intra-individual variation might impact the readings of serum magnesium and thus obscure the association between serum magnesium levels and depressive symptoms in women.

The current reference interval for serum magnesium was defined by measuring serum magnesium levels in healthy individuals taking part in the first National Health and Nutrition Examination Survey in 1974 (NHANES I 1974) [27]. The central 95th percentile of serum magnesium level (0.75–0.95 mmol/L) of the included 15,820 subjects, aged 18–74, was defined as the normal range. It must be acknowledged that this reference interval is based on the distribution of serum magnesium in the population, but not the association between serum magnesium and health outcomes. A re-evaluation of this reference interval, based on new evidence with respect to health outcomes, has been advocated [30].

Chronic latent magnesium deficiency (CLMD) was defined as a subclinical condition that makes individuals more vulnerable to disease; meanwhile, the serum magnesium levels are above the lower cut-off point for the reference interval (0.75 mmol/L). Previous studies showed that CLMD was associated with a higher risk of disease [30,36,37,38]. CLMD is a consequence of a small chronic negative magnesium balance, which may be caused by decreased intake, decreased absorption, and/or increased excretion [24]. The most commonly noticed reason for CLMD is a decreased magnesium dietary intake, since processed and refined food tend to have a lower magnesium content [25,26]. Illness and drug use must also be taken into account. These reasons together contribute to a negative magnesium balance and cause the serum magnesium concentration to decrease. In response to this low-key chronic process, magnesium was shown to be depleted from bone to support the homeostasis of serum magnesium [36]. This equilibrating process is chronic because it extends over years, even throughout a lifetime. It is latent because the serum concentration level tested is within the reference interval, even at the lower end, and the examinee is thus categorized as having normal magnesium status. Therefore, individuals with CLMD appear to have “normal” test results, but they do not maintain a magnesium status sufficient for long-term health [36]. An evidence-based reference interval for serum magnesium is similar to what has been developed for cholesterol [39]. The previous reference interval for cholesterol was established from the statistics of cholesterol levels in normal individuals and had a large variance, especially in the samples from hospitals, with some having an upper cutoff > 300 mg/dL. After reviewing the medical literature, an evidence-based upper limit of the reference interval for serum total cholesterol was proposed by a consensus conference held at the National Institute of Health; this value was 200 mg/dL. Emerging evidence has supported a similar revision of the reference interval for serum magnesium [30,36,37,38]. Experts in magnesium research, one group from the United States and another from Germany, have proposed a serum magnesium value of 0.85 mmol/L as the low cut-off point to define hypomagnesemia; collected data suggested an increased risk of cardiovascular diseases, diabetes, and mortality from these diseases, even with values between 0.75 and 0.85 mmol/L [29,30]. The international group of magnesium researchers (Magnesium Global Network [MaGNet]) further recommends an updated standardization of the serum magnesium reference range based on their survey of reference range values from collaborating institutions, hospitals, and colleagues worldwide [28]. The evidence-based new cut-off value of 0.85 mmol/L prevents the inclusion of individuals with CLMD, who usually fall into the lower half of the reference interval (0.75–0.85 mmol/L) and tend to be misclassified as having a normal magnesium status.

Our results, in line with previous studies, present the correlation of CLMD with depressive symptoms. Symptoms did not differ between those with CLMD and those with hypomagnesemia in either linear or logistic analysis. However, those with serum magnesium ≥ 0.85 mmol/L showed significantly lower depressive scores than those with hypomagnesemia. This is the first analysis focusing on the association between CLMD and depressive symptoms, and suggests that CLMD is also applicable to mental disorders. Whether this association also exists with respect to other psychological symptoms or major mental disorders requires further study. Previous study also indicated the magnesium deficits in depression may share similar pathway with other psychiatric disorders and cognitive decline [40]. Our study may also raise the awareness of mental health professionals regarding the provision of necessary dietary and therapeutic interventions, in order to meet target magnesium concentrations, thereby decreasing the risk of developing depressive symptoms and other adverse health outcomes caused by CLMD.

This study has some specific strengths. First, our data are nationally representative of Taiwanese community-dwelling adults. Second, we adjusted for potential confounders related to the outcome. Third, we report serum magnesium and dietary magnesium intake simultaneously; this offers better opportunities for differentiating their effects on depressive symptoms. In addition, we compared the symptom severity and odds ratio among different serum magnesium level stratifications, in order to present a dose effect of magnesium status on depressive symptoms.

The main implication of our results is that low serum magnesium levels confer an increased odds ratio for depressive symptoms in community-dwelling adults in Taiwan. Although depressive symptoms and depression are based on multifactorial mechanisms, our results raise the possibility that adequate functional magnesium status, along with the modification of other risk factors, might present a meaningful method in order to prevent depressive symptoms. Our findings suggest that dietary magnesium intake alone may be inadequate to achieve such an effect. However, whether a pharmacological dose of magnesium supply (with magnesium supplements or an aggressive dietary plan), in order to reach a targeted serum magnesium level, can reduce the risk of depression/the severity of depressive symptoms requires further investigation.

The study was also subject to some possible limitations. First, this and previous studies that have shown connections between serum magnesium and symptoms/illnesses were carried out only in ethnically homogenous populations; whether ethnic differences exist in the connection between serum magnesium and depressive symptoms needs further exploration. Second, serum magnesium levels vary during different phases of the menstruation cycle. Thus, the measurement of serum magnesium concentrations among female subjects might have been biased and led to a null result. Third, the evaluation of depressive symptoms was self-reported. Fourth, the study participants were relatively healthy subjects living in the community, who might present different characteristics from patients with major depressive disorder or other major physical illness. The relationship between magnesium status and major depressive disorder needs further examination. Fifth, the character of this research, as an association study, impedes further inference of causality. Given that magnesium status is dynamic with daily changes, the time window of the association requires further exploration. Sixth, serum magnesium cannot represent total body magnesium storage because it only makes up 1% of total body magnesium. However, a consensus on a gold standard to represent the functionality of magnesium is absent, and based on the correlation of serum magnesium with the intracellular magnesium level, the associations described here might well be underestimated [41]. Seventh, our study did not analyze supplemental magnesium separately from other forms of magnesium intake. Thus, we could not investigate the specific effect of a pharmacological dose of supplemental magnesium; this limits the externalization of our results regarding the effect of magnesium supplement against depressive symptoms. Lastly, the presence of residual confounders cannot be ruled out. For example, factors influencing magnesium absorption and excretion after dietary intakes, such as calcium intake and medication, could not be completely controlled for in the analysis [42].

## 5. Conclusions

In conclusion, serum magnesium was negatively associated with depressive symptoms. Subjects with CLMD exhibited a similar risk and symptom severity to those with magnesium deficiency, defined by the current threshold. The relationship between oral magnesium intake, both from dietary and magnesium supplements, and depressive symptoms, requires further investigation. Since magnesium status is dynamic, its effect on depressive symptoms over longer periods of time also requires further examination.

## Figures and Tables

**Table 1:** Participant characteristics (N = 2193).

	Mean (±SD) or N (%)
Sex	
Women	1124 (51.25)
Men	1069 (48.75)
Education level	
None	208 (9.48)
Below SHS	934 (42.59)
SHS	585 (26.68)
Above SHS	466 (21.25)
Income level	
No income	533 (24.30)
NTD 20,000 or lower	839 (38.26)
NTD 20,001 to 50,000	569 (25.95)
NTD 50,001 to 70,000	153 (6.98)
NTD 70,001 or greater	99 (4.51)
Physical activity status	
No	977 (44.55)
Yes—walking	613 (27.95)
Yes—more than walking^5^	603 (27.50)
Alcohol use	
Non-drinker	1183 (53.94)
Former drinker	178 (8.12)
Current drinker	832 (37.94)
Smoking status	
Non-smoker	1490 (67.94)
Former smoker	272 (12.40)
Current smoker	431 (19.65)
CRP (mg/L)	
CRP < 1	1041 (47.47)
1 ≤ CRP < 3	754 (34.38)
CRP ≥ 3	398 (18.15)
DM	
No	2005 (91.43)
Yes	188 (8.57)
Hypertension	
No	1665 (75.92)
Yes	528 (24.08)
Kidney disease	
No	2153 (98.18)
Yes	40 (1.82)
Age (per year)	
	53.36 (±17.36)
Dietary Mg (mg/day)	
	278 (±185)
Serum Mg (mg/dL)	
	2.14 (±0.19)
Dietary Ca (mg/day)	
	611 (±527)
Serum Ca (mg/dL)	
	9.18 (±0.43)
Total energy intake	
	1.86 (±0.96)
BSRS—Insomnia	
	0.62 (±1.02)
BSRS—Anxiety	
	0.29 (±0.66)
BSRS—Hostility	
	0.37 (±0.71)
BSRS—Depression	
	0.35 (±0.69)
BSRS—Inferiority	
	0.23 (±0.60)
BSRS Total score	
	1.86 (±2.6)

BSRS = Brief Symptom Rating Scale; CRP = C-reactive protein; DM = Diabetes mellitus; NTD = New Taiwan dollar; SD = Standard deviation; SE = Standard error; SHS = Senior high school; Mg = Magnesium. Unit of total energy intake was in 1000 kcal.

**Table 2:** Simple and multiple linear regression analysis of BSRS-5 outcome measures versus predictor variables (dietary or serum magnesium).

	Serum Mg			Dietary Mg ^1^		
Outcome	β	*p*-Value	95% CI	β	*p*-Value	95% CI
BSRS score						
Simple model	−0.85	0.0041 **	(−1.43, −0.27)	−0.02	0.5685	(−0.08, 0.04)
Model 1 ^2^	−0.43	0.1463	(−1.02, 0.15)	−0.01	0.8338	(−0.09, 0.07)
Model 2 ^3^	−0.48	0.1142	(−1.07, 0.11)	−0.01	0.8073	(−0.11, 0.08)
Insomnia						
Simple model	−0.26	0.0262 *	(−0.48, −0.03)	−0.01	0.3857	(−0.03, 0.01)
Model 1 ^2^	−0.10	0.4119	(−0.33, 0.14)	−0.01	0.6749	(−0.04, 0.02)
Model 2 ^3^	−0.13	0.2899	(−0.36, 0.11)	−0.01	0.5761	(−0.05, 0.03)
Anxiety						
Simple model	−0.05	0.5051	(−0.20, 0.10)	0.00	0.7923	(−0.01, 0.02)
Model 1 ^2^	−0.04	0.6443	(−0.19, 0.12)	0.01	0.2116	(−0.01, 0.03)
Model 2 ^3^	−0.04	0.6287	(−0.19, 0.11)	0.02	0.5761	(−0.00, 0.05)
Hostility						
Simple model	−0.11	0.1595	(−0.27, 0.05)	0.00	0.5616	(−0.01, 0.02)
Model 1 ^2^	−0.04	0.6325	(−0.20, 0.12)	0.01	0.6341	(−0.02, 0.03)
Model 2 ^3^	−0.04	0.6404	(−0.20, 0.12)	−0.00	0.9433	(−0.03, 0.02)
Depression						
Simple model	−0.26	0.0011 **	(−0.41, −0.10)	−0.01	0.1431	(−0.03, 0.00)
Model 1 ^2^	−0.18	0.0239 *	(−0.34, −0.02)	−0.01	0.2058	(−0.04, 0.01)
Model 2 ^3^	−0.16	0.0226 *	(−0.34, −0.03)	−0.01	0.3044	(−0.04, 0.01)
Inferiority						
Simple model	−0.17	0.0127 *	(−0.30, −0.04)	0.00	0.7720	(−0.02, 0.01)
Model 1 ^2^	−0.08	0.2405	(−0.22, 0.05)	−0.01	0.5030	(−0.02, 0.01)
Model 2 ^3^	−0.09	0.1997	(−0.23, 0.05)	−0.01	0.4338	(−0.03, 0.01)

BSRS = Brief Symptom Rating Scale; CI = Confidence interval; Mg = Magnesium; Ca = Calcium; * *p* < 0.05; ** *p* < 0.01. ^1^ Unit of dietary Mg was in 100 mg. ^2^ Adjusted for age, sex, educational levels, income levels, smoking status, alcohol use, physical activity status, hypertension, diabetes mellitus, kidney disease, c-reactive protein, and total energy intake (per 1000 kcal). ^3^ Adjusted for all variables in model 1 plus Ca.

**Table 3:** Simple and multiple logistic regression analysis of BSRS-5 outcome measures versus predictor variables (dietary or serum magnesium).

	Serum Mg			Dietary Mg ^1^		
Outcome	ORs	*p*-Value	95% CI	ORs	*p*-Value	95% CI
BSRS Total score						
Simple model	0.436	0.0337 *	(0.203, 0.938)	0.984	0.7066	(0.905, 1.070)
Adjusted model 1 ^2^	0.657	0.3101	(0.292,1.478)	1.017	0.7696	(0.908, 1.139)
Adjusted model 2 ^3^	0.714	0.4184	(0.316, 1.614)	0.978	0.7558	(0.850, 1.125)
Insomnia						
Simple model	0.518	0.0285 *	(0.287, 0.933)	1.009	0.7630	(0.950, 1.072)
Adjusted model 1 ^2^	0.695	0.2430	(0.377, 1.280)	1.004	0.9271	(0.925, 1.090)
Adjusted model 2 ^3^	0.677	0.2145	(0.366, 1.254)	1.008	0.8669	(0.916, 1.109)
Anxiety						
Simple model	0.616	0.2867	(0.252, 1.502)	1.020	0.6537	(0.934, 1.115)
Adjusted model 1 ^2^	0.664	0.3985	(0.256, 1.719)	1.101	0.0864	(0.986, 1.228)
Adjusted model 2 ^3^	0.710	0.4861	(0.271, 1.861)	1.129	0.0550 ^†^	(0.997, 1.227)
Hostility						
Simple model	0.621	0.2524	(0.275, 1.404)	1.071	0.0626	(0.996, 1.151)
Adjusted model 1 ^2^	0.767	0.5525	(0.319, 1.843)	1.085	0.1117	(0.981, 1.200)
Adjusted model 2 ^3^	0.809	0.6402	(0.333, 1.967)	1.061	0.3497	(0.938, 1.200)
Depression						
Simple model	0.315	0.0074 **	(0.135, 0.733)	0.921	0.1317	(0.828, 1.025)
Adjusted model 1 ^2^	0.378	0.0330 *	(0.155, 0.924)	0.935	0.3808	(0.804, 1.087)
Adjusted model 2 ^3^	0.420	0.0594 ^†^	(0.170, 1.035)	0.873	0.1696	(0.719, 1.060)
Inferiority						
Simple model	0.213	0.0034 **	(0.076, 0.599)	0.955	0.4803	(0.841, 1.085)
Adjusted model 1 ^2^	0.305	0.0336 *	(0.102, 0.912)	0.911	0.3306	(0.755, 1.099)
Adjusted model 2 ^3^	0.405	0.1099	(0.134, 1.226)	0.836	0.1522	(0.654, 1.068)

BSRS = Brief Symptom Rating Scale; CI = Confidence interval; Mg = Magnesium; Ca = Calcium; * *p* < 0.05; ** *p* < 0.01; † marginally significant. ^1^ Unit of dietary Mg was in 100 mg.^2^ Adjusted for age, sex, educational levels, income levels, smoking status, alcohol use, physical activity status, hypertension, diabetes mellitus, kidney disease, c-reactive protein, and total energy intake (per 1000 kcal). ^3^ Adjusted for all variables in model 1 plus Ca.

**Table 4:** Simple and multiple linear regression analysis of BSRS-5 outcome measures versus predictor variables (serum magnesium) by sex.

	Men			Women		
Outcome	β	*p*-Value	95% CI	β	*p*-Value	95% CI
BSRS Total score						
Simple model	−1.79	< 0.0001 ***	(−2.58, −0.99)	0.18	0.6774	(−0.65, 1.01)
Model 1 ^1^	−1.49	0.0004 ***	(−2.31, −0.67)	0.63	0.1394	(−0.21, 1.47)
Model 2 ^2^	−1.56	0.0002 ***	(−2.39, −0.73)	0.61	0.1578	(−0.23, 1.45)
Model 3 ^3^	-	-	-	0.53	0.2373	(−0.35, 1.40)
Insomnia						
Simple model	−0.76	< 0.0001 ***	(−1.07, −0.45)	0.28	0.0957	(−0.05, 0.60)
Model 1 ^1^	−0.61	0.0002 ***	(−0.93, −0.28)	0.39	0.0217 *	(0.06, 0.72)
Model 2 ^2^	−0.64	0.0001 ***	(−0.97, −0.32)	0.38	0.0276 *	(0.04, 0.71)
Model 3 ^3^	-	-	-	0.35	0.0482 *	(0.00, 0.69)
Anxiety						
Simple model	−0.25	0.0057 **	(−0.42, −0.07)	0.17	0.1509	(−0.06, 0.41)
Model 1 ^1^	−0.25	0.0078 **	(−0.43, −0.07)	0.18	0.1515	(−0.06, 0.42)
Model 2 ^2^	−0.25	0.0077 **	(−0.43, −0.07)	0.17	0.1595	(−0.07, 0.42)
Model 3 ^3^	-	-	-	0.15	0.2362	(−0.10, 0.40)
Hostility						
Simple model	−0.16	0.1503	(−0.38, 0.06)	−0.06	0.6285	(−0.29, 0.17)
Model 1 ^1^	−0.13	0.2724	(−0.35, 0.10)	0.07	0.5743	(−0.16, 0.29)
Model 2 ^2^	−0.13	0.2691	(−0.35, 0.10)	0.06	0.5981	(−0.17, 0.29)
Model 3 ^3^	-	-	-	0.06	0.5956	(−0.17, 0.30)
Depression						
Simple model	−0.39	0.0004 ***	(−0.60, −0.18)	−0.11	0.3100	(−0.34, 0.11)
Model 1 ^1^	−0.35	0.0019 **	(−0.58, −0.13)	0.00	0.9929	(−0.23, 0.22)
Model 2 ^2^	−0.36	0.0018 **	(−0.59, −0.13)	−0.00	0.9835	(−0.23, 0.22)
Model 3 ^3^	-	-	-	−0.01	0.9388	(−0.24, 0.23)
Inferiority						
Simple model	−0.23	0.0133 *	(−0.42, −0.05)	−0.10	0.3011	(−0.29, 0.09)
Model 1 ^1^	−0.16	0.1048	(−0.35, 0.03)	0.00	0.9959	(−0.20, 0.20)
Model 2 ^2^	−0.17	0.0733	(−0.37, 0.02)	−0.00	0.9683	(−0.20, 0.19)
Model 3 ^3^	-	-	-	−0.03	0.7990	(−0.23, 0.18)

BSRS = Brief Symptom Rating Scale; CI = Confidence interval; Mg = Magnesium; * *p* < 0.05; ** *p* < 0.01; *** *p* < 0.001. ^1^ Adjusted for age, educational levels, income levels, smoking status, alcohol use, physical activity status, hypertension, diabetes mellitus, kidney disease, c-reactive protein, and total energy intake (per 1000 kcal). ^2^ Adjusted for variables in model 1 plus Ca.^3^ Adjusted for variables in model 2 plus menopause.

## Data Availability

Not applicable.

## References

[1] World Health Organization (2008). World Health Statistics 2008.

[2] Vos T., Lim S.S., Abbafati C., Abbas K.M., Abbasi M., Abbasifard M., Abbasi-Kangevari M., Abbastabar H., Abd-Allah F., Abdelalim A.J. (2020). Global burden of 369 diseases and injuries in 204 countries and territories, 1990–2019: A systematic analysis for the Global Burden of Disease Study 2019. Lancet.

[3] Mokdad A.H., Ballestros K., Echko M., Glenn S., Olsen H.E., Mullany E., Lee A., Khan A.R., Ahmadi A., Ferrari A.J. (2018). The state of US health, 1990–2016: Burden of diseases, injuries, and risk factors among US states. JAMA.

[4] Ferrari A.J., Charlson F.J., Norman R.E., Patten S.B., Freedman G., Murray C.J., Vos T., Whiteford H.A. (2013). Burden of depressive disorders by country, sex, age, and year: Findings from the global burden of disease study 2010. PLoS Med..

[5] Pickering G., Mazur A., Trousselard M., Bienkowski P., Yaltsewa N., Amessou M., Noah L., Pouteau E. (2020). Magnesium status and stress: The vicious circle concept revisited. Nutrients.

[6] Newport D.J., Carpenter L.L., McDonald W.M., Potash J.B., Tohen M., Nemeroff C.B., APA Council of Research Task Force on Novel Biomarkers and Treatments (2015). Ketamine and other NMDA antagonists: Early clinical trials and possible mechanisms in depression. Am. J. Psychiatry.

[7] Lang U.E., Beglinger C., Schweinfurth N., Walter M., Borgwardt S. (2015). Nutritional aspects of depression. Cell. Physiol. Biochem..

[8] Eby G.A., Eby K.L. (2010). Magnesium for treatment-resistant depression: A review and hypothesis. Med. Hypotheses.

[9] Fang X., Wang K., Han D., He X., Wei J., Zhao L., Imam M.U., Ping Z., Li Y., Xu Y. (2016). Dietary magnesium intake and the risk of cardiovascular disease, type 2 diabetes, and all-cause mortality: A dose–response meta-analysis of prospective cohort studies. BMC Med..

[10] Han H., Fang X., Wei X., Liu Y., Jin Z., Chen Q., Fan Z., Aaseth J., Hiyoshi A., He J. (2017). Dose-response relationship between dietary magnesium intake, serum magnesium concentration and risk of hypertension: A systematic review and meta-analysis of prospective cohort studies. Nutr. J..

[11] Zamani M., Haghighat N. (2022). The Effects of Magnesium Supplementation on Serum Magnesium and Calcium Concentration in Patients With Type 2 Diabetes: A Systematic Review and Meta-Analysis of Randomized Controlled Trials. Clin. Nutr. Res..

[12] Li B., Lv J., Wang W., Zhang D.J.A., Psychiatry N.Z. (2017). Dietary magnesium and calcium intake and risk of depression in the general population: A meta-analysis. Aust. N. Z. J. Psychiatry.

[13] Tarleton E.K., Littenberg B. (2015). Magnesium intake and depression in adults. J. Am. Board Fam. Med..

[14] Sun C., Wang R., Li Z., Zhang D. (2019). Dietary magnesium intake and risk of depression. J. Affect. Disord..

[15] Asbaghi O., Moradi S., Kashkooli S., Zobeiri M., Nezamoleslami S., Lazaridi A.-V., Miraghajani M. (2022). The effects of oral magnesium supplementation on glycemic control in patients with type 2 diabetes: A systematic review and dose-response meta-analysis of controlled clinical trials. Br. J. Nutr..

[16] Workinger J.L., Doyle R.P., Bortz J. (2018). Challenges in the Diagnosis of Magnesium Status. Nutrients.

[17] Szkup M., Jurczak A., Brodowska A., Brodowska A., Noceń I., Chlubek D., Laszczyńska M., Karakiewicz B., Grochans E. (2017). Analysis of relations between the level of Mg, Zn, Ca, Cu, and Fe and depressiveness in postmenopausal women. Biol. Trace Elem. Res..

[18] Islam M.R., Islam M.R., Shalahuddin Qusar M., Islam M.S., Kabir M.H., Mustafizur Rahman G., Islam M.S., Hasnat A. (2018). Alterations of serum macro-minerals and trace elements are associated with major depressive disorder: A case-control study. BMC Psychiatry.

[19] Tarleton E.K., Kennedy A.G., Rose G.L., Crocker A., Littenberg B. (2019). The association between serum magnesium levels and depression in an adult primary care population. Nutrients.

[20] Botturi A., Ciappolino V., Delvecchio G., Boscutti A., Viscardi B., Brambilla P. (2020). The role and the effect of magnesium in mental disorders: A systematic review. Nutrients.

[21] Kao W.H.L., Folsom A.R., Nieto F.J., Mo J.-P., Watson R.L., Brancati F.L. (1999). Serum and Dietary Magnesium and the Risk for Type 2 Diabetes Mellitus: The Atherosclerosis Risk in Communities Study. Arch. Intern. Med..

[22] Wei J., Zeng C., Li X.X., Gong Q.Y., Lei G.H., Yang T.B. (2016). Association among dietary magnesium, serum magnesium, and diabetes: A cross-sectional study in middle-aged and older adults. J. Health Popul. Nutr..

[23] Peacock J.M., Folsom A.R., Arnett D.K., Eckfeldt J.H., Szklo M. (1999). Relationship of Serum and Dietary Magnesium to Incident Hypertension: The Atherosclerosis Risk in Communities (ARIC) Study. Ann. Epidemiol..

[24] Reddy S.T., Soman S.S., Yee J. (2018). Magnesium balance and measurement. Adv. Chronic Kidney Dis..

[25] Sawicki C.M., Jacques P.F., Lichtenstein A.H., Rogers G.T., Ma J., Saltzman E., McKeown N.M. (2021). Whole-and refined-grain consumption and longitudinal changes in cardiometabolic risk factors in the framingham offspring cohort. J. Nutr..

[26] Rosanoff A. (2013). Changing crop magnesium concentrations: Impact on human health. Plant Soil.

[27] Lowenstein F.W., Stanton M.F. (1986). Serum magnesium levels in the United States, 1971–1974. J. Am. Coll. Nutr..

[28] Rosanoff A., West C., Elin R.J., Micke O., Baniasadi S., Barbagallo M., Campbell E., Cheng F.C., Costello R.B., Gamboa-Gomez C. (2022). Recommendation on an updated standardization of serum magnesium reference ranges. Eur. J. Nutr..

[29] Micke O., Vormann J., Kraus A., Kisters K. (2021). Serum magnesium: Time for a standardized and evidence-based reference range. Magnes. Res..

[30] Costello R.B., Elin R.J., Rosanoff A., Wallace T.C., Guerrero-Romero F., Hruby A., Lutsey P.L., Nielsen F.H., Rodriguez-Moran M., Song Y. (2016). Perspective: The case for an evidence-based reference interval for serum magnesium: The time has come. Adv. Nutr..

[31] Tu S.H., Chen C., Hsieh Y.T., Chang H.Y., Yeh C.J., Lin Y.C., Pan W.H. (2011). Design and sample characteristics of the 2005-2008 Nutrition and Health Survey in Taiwan. Asia Pac. J. Clin. Nutr..

[32] Pan W.-H., Chang Y.-H., Chen J.-Y., Wu S.-J., Tzeng M.-S., Kao M.-D. (1999). Nutrition and Health Survey in Taiwan (NAHSIT) 1993-1996: Dietary Nutrient Intakes Assessed by 24-Hour Recall. Nutr. Sci. J..

[33] Ma J., Folsom A.R., Melnick S.L., Eckfeldt J.H., Sharrett A.R., Nabulsi A.A., Hutchinson R.G., Metcalf P.A. (1995). Associations of serum and dietary magnesium with cardiovascular disease, hypertension, diabetes, insulin, and carotid arterial wall thickness: The ARIC study. Atherosclerosis Risk in Communities Study. J. Clin. Epidemiol..

[34] Seeman M.V. (1997). Psychopathology in women and men: Focus on female hormones. Am. J. Psychiatry.

[35] Dullo P., Vedi N. (2008). Changes in serum calcium, magnesium and inorganic phosphorus levels during different phases of the menstrual cycle. J. Hum. Reprod. Sci..

[36] Elin R.J. (2010). Assessment of magnesium status for diagnosis and therapy. Magnes. Res..

[37] Nielsen F.H., Johnson L.A.K. (2017). Data from controlled metabolic ward studies provide guidance for the determination of status indicators and dietary requirements for magnesium. Biol. Trace Elem. Res..

[38] Nielsen F.H. (2016). Guidance for the determination of status indicators and dietary requirements for magnesium. Magnes. Res..

[39] National Heart, Lung, and Blood Institute (1985). Consensus Conference: Lowering blood cholesterol to prevent heart disease. JAMA.

[40] Barbagallo M., Veronese N., Dominguez L.J. (2021). Magnesium in Aging, Health and Diseases. Nutrients.

[41] Ryzen E., Servis K., DeRusso P., Kershaw A., Stephen T., Rude R.K. (1989). Determination of intracellular free magnesium by nuclear magnetic resonance in human magnesium deficiency. J. Am. Coll. Nutr..

[42] Chonan O., Takahashi R., Yasui H., Watanuki M. (1997). The effect of calcium gluconate and other calcium supplements as a dietary calcium source on magnesium absorption in rats. Int. J. Vitam. Nutr. Res..

